# Auricular Electrical Stimulation Alleviates Headache through CGRP/COX-2/TRPV1/TRPA1 Signaling Pathways in a Nitroglycerin-Induced Migraine Rat Model

**DOI:** 10.1155/2019/2413919

**Published:** 2019-12-11

**Authors:** Chung-Chih Liao, Jung-Miao Li, Ching-Liang Hsieh

**Affiliations:** ^1^Graduate Institute of Chinese Medicine, College of Chinese Medicine, China Medical University, Taichung 40402, Taiwan; ^2^Department of Chinese Medicine, Show Chwan Memorial Hospital, Changhua 50008, Taiwan; ^3^Chinese Medicine Research Center, China Medical University, Taichung 40402, Taiwan; ^4^Graduate Institute of Acupuncture Science, College of Chinese Medicine, China Medical University, Taichung 40402, Taiwan; ^5^Department of Chinese Medicine, China Medical University Hospital, Taichung 40447, Taiwan

## Abstract

The study aimed to investigate effect of auricular electrical stimulation (ES) on migraine. Migraine was induced in rats by intraperitoneal administration of nitroglycerin (NTG, 10 mg/kg) three times. Auricular ES pretreatment was performed for five consecutive days. Migraine behaviors were observed by a video recording. Auricular ES pretreatment could reverse the decrease of the total time spent on exploratory (2619.0 ± 113.0 s vs 1581.7 ± 217.6 s, *p*=0.0029) and locomotor behaviors (271.3 ± 21.4 s vs 114.3 ± 19.7 s, *p*=0.0135) and also could reverse the increase of the total time spent on resting (19.0 ± 10.6 s vs 154.3 ± 46.5 s, *p*=0.0398) and grooming (369.9 ± 66.8 s vs 1302.0 ± 244.5 s, *p*=0.0324) behaviors. Auricular ES pretreatment could increase the frequency of rearing behaviors (38.0 ± 1.8 vs 7.7 ± 3.5, *p* < 0.0001) and total distance traveled (1372.0 ± 157.9 cm vs 285.3 ± 85.6 cm, *p* < 0.0001) and also could increase the percentage of inner zone time (6.0 ± 1.6% vs 0.4 ± 0.2%, *p*=0.0472). The CGRP, COX-2, TRPV1, and TRPA1 immunoreactive cells in the trigeminal ganglion increased in the NTG group compared with the control group (all *p* < 0.0001); this increase could, however, be reduced by auricular ES pretreatment (27.8 ± 2.6 vs 63.0 ± 4.2, *p* < 0.0001; 21.7 ± 1.2 vs 61.8 ± 4.0, *p* < 0.0001; 24.3 ± 1.0 vs 36.5 ± 1.7, *p*=0.0003; and 20.7 ± 1.9 vs 90.8 ± 6.5, *p* < 0.0001, respectively). Therefore, we suggest that auricular ES pretreatment is beneficial for the treatment of migraine and this effect is partly related to CGRP/COX-2/TRPV1/TRPA1 signaling pathways.

## 1. Introduction

Migraine is a common neurological disease that is characterized by unilateral, pulsating, moderate to severe pain and is associated with nausea, photophobia, or phonophobia [1]. Notably, migraine is the sixth most prevalent and second most disabling disease in the world [2]. Migraine often affects a patient's routine activities and imposes substantial social and financial burdens [3, 4]. Psychiatric disorders have a high prevalence worldwide and are a leading cause of disability and death. Depression and anxiety are the most frequent psychiatric disorders associated with migraine, especially in chronic migraineurs [5]. A study reported that migraineurs have a more than 2.5-fold higher risk of developing depression compared with healthy individuals; moreover, anxiety disorders are two to five times more prevalent in migraineurs than in healthy individuals [6].

Prophylactic medications are often prescribed to migraineurs with more frequent or more painful migraine attacks compared with other individuals. However, prophylactic drugs have an average efficacy rate of less than 50% and may engender intolerable adverse effects [7]. Hence, numerous nonpharmacological treatments have been increasingly developed in recent years, such as peripheral nerve or transcranial neurostimulation [7, 8].

Acupuncture has been practiced throughout Asia for thousands of years. The analgesic effect of acupuncture is widely approved in basic and clinical science worldwide. Reports have proven that acupuncture is an effective therapy for reducing pain severity in acute migraine attacks or preventing frequent and chronic migraine attacks [9, 10]. Auricular acupuncture—a major routine acupuncture practice—has been employed to treat neurological diseases [11]. The auricular sensory afferent is primarily innervated by the trigeminal, facial, glossopharyngeal, and vagus nerves, all of which have parasympathetic activity except for the trigeminal nerve [12]. Vagus nerve stimulation (VNS) has been proven to be effective for the mitigation of acute migraine or in the preventive treatment of migraine [13, 14]. Because the skin around the ears is enriched with branches of the vagus nerve, auricular acupuncture has been hypothesized to be an effective, convenient, and safe alternative for VNS therapy. Our previous studies on rodents have reported that auricular electrical stimulation (ES) could activate parasympathetic tone when applied to treat or ameliorate inflammatory diseases, such as epilepsy [15, 16], ischemic stroke [17], and obesity [18]. Therefore, the mechanism underlying the effects of auricular ES on prevention of migraine and psychiatric disorders is a notable and appealing topic.

In human and experimental models, systemic administration of nitroglycerin (NTG) has been extensively performed to induce delayed response of migraine-like symptoms through trigeminovascular system activation. Repeated injections of NTG in rats can adequately mimic frequent migraine or chronic migraine; such injections can result in a more effective model for testing the efficacy of migraine prevention therapy when compared with a single dose of NTG injection [19, 20]. Nociceptive signals from meningeal blood vessels through primary afferent nerve fibers connect to the trigeminal ganglion (TG) neurons and then transmit to the central terminals in the trigeminal nucleus caudalis (TNC) [21]. Therefore, the excitability of TG neurons through peripheral sensitization is considered a crucial factor in migraine pathogenesis [22].

The aim of the present study was to investigate the effects of auricular ES on migraine and associated psychiatric symptoms; the study also explored the mechanisms underlying such effects. We established a migraine rat model by repeatedly administering NTG intraperitoneally.

## 2. Materials and Methods

### 2.1. Animals

This study used male Sprague-Dawley (SD) rats weighing 200–300 g. The rats were purchased from BioLASCO (Taipei, Taiwan) and were maintained in a controlled environment with a 12/12 h light-dark cycle. The relative humidity was controlled at 55% ± 5%, and the room temperature was controlled at 23 ± 1°C. Food and tap water were provided *ad libitum.* Animal use was approved by the Institutional Animal Care and Use Committee (IACUC) of Show Chwan Memorial Hospital (No. 105031) and followed the Guide for the Use of Laboratory Animals (National Academy Press).

### 2.2. Establishment of the Migraine Rat Model

**Figure 1:**
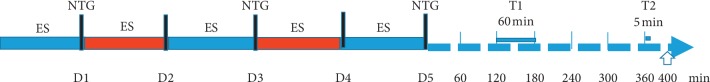
Experimental procedure. ES: auricular electrical stimulation (ES) pretreatment; NTG: nitroglycerin (10 mg/kg) intraperitoneal (i.p.) injection; D1: first day; D2: second day; D3: third day; D4: fourth day; D5: fifth day; T1, 60 min: rat behavior recognition module video recordings for 60 min; T2, 5 min: open-field test recordings for 5 min; the rats were euthanized 400 min after i.p. NTG injection on the fifth day (arrow head).

A migraine rat model was established by administering repeated intraperitoneal (IP) injections of NTG (10 mg/kg, Millisrol®; Nippon Kayaku, Co., Ltd. Japan) on three alternate days, namely, the first, third, and fifth days (Figure 1), which was referenced and modified from previous reports [19, 23] and our previous study [24].

### 2.3. Grouping

A total of 24 rats were randomly divided equally into four groups (six rats per group). For the first group (control group), the rats received IP injections of 0.9% saline solution on three alternate days continually. In the second group (NTG group), the rats received only IP injections of NTG (10 mg/kg) on three alternate days continually. In the third group (auricular ES group, also referred to as ES group), the rats received the same treatment as those in the NTG group; in addition, ES (frequency = 2 Hz; duration = 100 *μ*s) was applied to the ears (through the use of clip electrodes, with the cathode at the apex and anode at the lobe) by using an ES apparatus (Trio 300, Ito, Japan). The ES intensity was indicated by a visual ear twitch and was maintained for 20 min/day (10 min for each ear alternately) on days 2 and 4; ES pretreatment was applied 30 min prior to NTG administration on days 1, 3, and 5 in order to prevent ES interfere NTG-induced migraine behaviors (Figure 1). Finally, in the fourth group (sham group), the rats received the same treatments as those in the ES group, but the clip electrodes were connected to an ES apparatus without electric charge.

### 2.4. Rat Behavior Test in the Migraine Model

#### 2.4.1. Assessment of Spontaneous Nociceptive Behaviors

The rats were placed in a transparent acrylic apparatus (45 cm × 45 cm × 35 cm) 120 min after IP NTG administration corresponding to migraine behavior [24, 25] on the fifth day. A video camera (Panasonic WV-CP300) was placed 1 m in front of the apparatus, and the behavior of the rats was subsequently recorded for 60 min (Figure 1). The video recordings were analyzed automatically by using EthoVision XT 12.0 software (Noldus Information Technology, Leesburg, VA), which is an automated video tracking and motion analysis system. The software's behavior recognition module was employed for analyzing the rats' behavior. The spontaneous nociceptive behavior of the rats was classified into the following categories: (1) exploratory behavior, involving rearing (the rat stands in an upright posture including the rise and descend) and sniffing (the rat makes slight movements of the head, possibly with slight, discontinuous body displacement including sniffing the air, the wall, or the floor); (2) locomotor behavior, involving walking (the rat moves to another place, and hind legs move as well) and jumping (the rat moves quickly forward with both hind limbs at the same time. Jumping interrupts other states like walking); (3) resting behavior (the rat rests with hardly any movement; either sits or lies down, which includes sleeping. Apparently, no interest in the environment); and (4) grooming behavior (the rat grooms snout, head, fur, or genitals including scratching and licking of paws during a grooming session) [26–29]. The total time spent engaging in the behavior was calculated in the present study.

#### 2.4.2. Assessment of Depressive or Anxiety-Like Behaviors

Depressive or anxiety-like behaviors were assessed using an open-field apparatus 360 min after IP NTG administration so that the interpretation of depression and anxiety behavior on the fifth day is not affected by migraine behavior. The rats were placed in the center of a 60 cm × 60 cm × 60 cm opaque open-field apparatus with a black floor; the rats were allowed to explore the space freely. The rats' behaviors were videotaped for 5 min (Figure 1). In this study, the central 30 cm × 30 cm area of the apparatus was defined as the inner zone. In addition, the frequency of rearing behavior and the total distance traveled were defined as constituting an index of depression. The percentage of time spent in the inner zone (IT%, calculated as the time in the inner zone/300 s × 100) and the percentage of distance traveled in the inner zone (ID%, calculated as the inner zone distance/total traveling distance × 100) were also defined as constituting an index of anxiety. A detailed description of the analysis of such behaviors was provided previously [24, 30]. These behaviors were also analyzed using EthoVision XT 12.0 software. Mean heat maps for the rat groups were also plotted using EthoVision XT 12.0 software in order to analyze the behavior of the rats in each group.

### 2.5. Immunohistochemistry Staining

All rats were heavily anesthetized with isoflurane at 400 min after IP administration of NTG or normal saline on the fifth day. The rats were then transcardially perfused with normal saline, followed by 4% paraformaldehyde (Merck, Frankfurt, Germany) in 0.1 M phosphate-buffered saline (pH 7.4). The rats' TG tissues were removed and postfixed overnight for immunohistochemistry (IHC) staining.

In the IHC staining process, TG tissue sections collected from the rats in the different groups were processed. Consecutive sections of neutral-buffered 10% formalin-fixed and wax-embedded tissues were cut at a thickness of 3 *μ*m, mounted on coated slides, and then dried for 1.5 h at 56°C. IHC analysis was performed using a TA link mouse/rabbit polymer detection system (TAHC04D, BioTnA, Kaohsiung, Taiwan). Sections were deparaffinized and rehydrated through a descending series of graded alcohol. The calcitonin gene-related polypeptide (CGRP) and cyclooxygenase-2 (COX-2) antigen were unmasked by immersing the slides into a boiling Tris EDTA buffer (pH 9.0) and incubating them for 20 min. The retrieval of the transient receptor potential cation channel subfamily V member 1 (TRPV1) and transient receptor potential cation channel subfamily A member 1 (TRPA1) antigen was performed by treating the slides in citrate buffer (pH 6.0) and boiling them for 20 min. The slides were gradually cooled down for 15 min. Peroxidase activity in the tissues was then blocked with a peroxidase blocker (TAHC04D, BioTnA, Kaohsiung, Taiwan) for 15 min. After being washed with 0.05% Tween 20-PBS (PBST), the slides were incubated with immunoblock for at least 30 min to reduce nonspecific background staining. The slides were washed in PBST three times for 2 min and subsequently incubated with primary antibody diluent of rabbit anti-CGRP (ab47027, Abcam), rabbit anti-COX2 (bs-0732R, Bioss), rabbit anti-TRPV1 antibody (ACC-030, Alomone), and rabbit anti-TRPA1 (bs71291, Bioworld) at dilution ratios of 1 : 200, 1 : 500, 1 : 200, and 1 : 200, respectively, overnight at room temperature. The slides were rinsed three times with PBST at 2-min intervals and then reacted with the TAlink mouse/rabbit polymer detection system and DAB. Finally, the sections were counterstained with hematoxylin for 2 min and then rinsed with running tap water for 5 min. The slides were air dried and mounted using coverslips. Histological analysis was six slides in each group in each IHC stain.

TG images were recorded under a light microscope (BX51, Olympus, Japan) at 200x magnification. CGRP, COX-2, TRPV1, and TRPA1 immune positive staining were identified according to brown granules labeled in the soma of TG neurons. A skilled operator, blinded to the experiments, calculated the number of positive staining cells by using Image-Pro Plus 6.0 software (Media Cybernetics, Silver Spring, MD, USA).

### 2.6. Statistical Analysis

All data are presented as the mean ± standard error of the mean (mean ± SEM). Statistical significance among the control, NTG, ES, and sham groups was analyzed using one-way analysis of variance, followed by Tukey's post hoc test. A *p* value of <0.05 was considered statistically significant. GraphPad Prism 7.0 software (GraphPad Prism Software Inc., San Diego, CA, USA) was used for statistical analysis and data presentation.

## 3. Results

### 3.1. Effect of Auricular ES Pretreatment on Spontaneous Nociceptive Behaviors in NTG-Induced Migraine Rats

**Figure 2:**
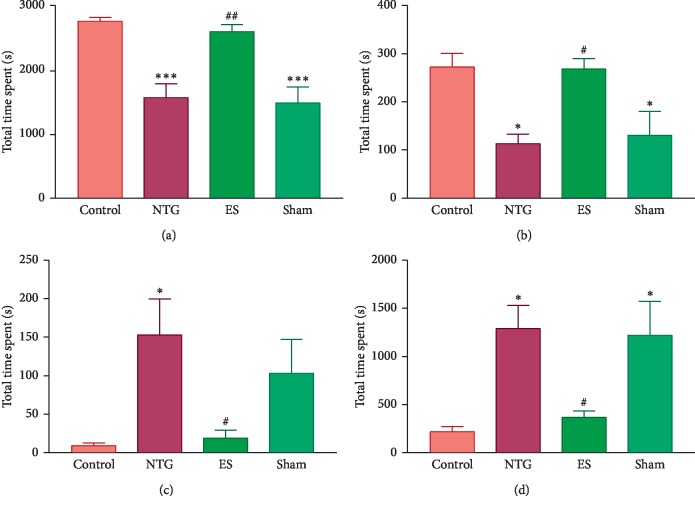
Effects of auricular electrical stimulation (ES) pretreatment on spontaneous nociceptive behaviors in the nitroglycerin (NTG)-induced migraine rat model: (a) total time spent on exploratory behavior; (b) total time spent on locomotor behavior; (c) total time spent on resting behavior; (d) total time spent on grooming behavior. Control: control group; NTG: NTG group; ES: ES group; sham: sham group. Data are presented as mean ± standard error of the mean. ^*∗*^*p* < 0.05, ^*∗∗*^*p* < 0.01, ^*∗∗∗*^*p* < 0.001 vs control group; ^#^*p* < 0.05, ^##^*p* < 0.01, ^###^*p* < 0.001 vs NTG group.

The total time spent on exploratory behavior, which involved rearing and sniffing, was significantly shorter in the NTG (1581.7 ± 217.6 s) and sham (1498.0 ± 253.2 s) groups than it was in the control (2784.6 ± 61.8 s) group (*p*=0.0006 and *p*=0.0003, respectively; Figure 2(a)). The total time spent was longer in the ES (2619.0 ± 113.0 s) group than it was in the NTG group (*p*=0.0029; Figure 2(a)).

The total time spent on locomotor behavior, which involved walking and jumping, was significantly shorter in the NTG (114.3 ± 19.7 s) and sham (131.6 ± 50.7 s) groups than it was in the control (274.9 ± 28.6 s) group (*p* = 0.0113 and *p*=0.0257, respectively; Figure 2(b)). The total time spent was longer in the ES (271.3 ± 21.4 s) group than it was in the NTG group (*p*=0.0135; Figure 2(b)).

The total time spent on resting behavior, which involved sleeping and hardly any movement, was significantly longer in the NTG (154.3 ± 46.5 s) group than it was in the control (9.2 ± 3.6 s) group (*p*=0.0255; Figure 2(c)). The total time spent was shorter in the ES (19.0 ± 10.6 s) group than it was in the NTG group (*p*=0.0398; Figure 2(c)).

Total time spent on grooming behavior, which involved body grooming, scratching, and licking of paws, was significantly longer in the NTG (1302.0 ± 244.5 s) and sham groups (1232.0 ± 353.4 s) than it was in the control (221.4 ± 53.6 s) group (*p*=0.0114 and *p*=0.0188, respectively; Figure 2(d)). The total time spent was shorter in the ES (369.9 ± 66.8 s) group than it was in the NTG group (*p*=0.0324; Figure 2(d)).

### 3.2. Effect of Auricular ES Treatment on Depression or Anxiety in NTG-Induced Migraine Rats

**Figure 3:**
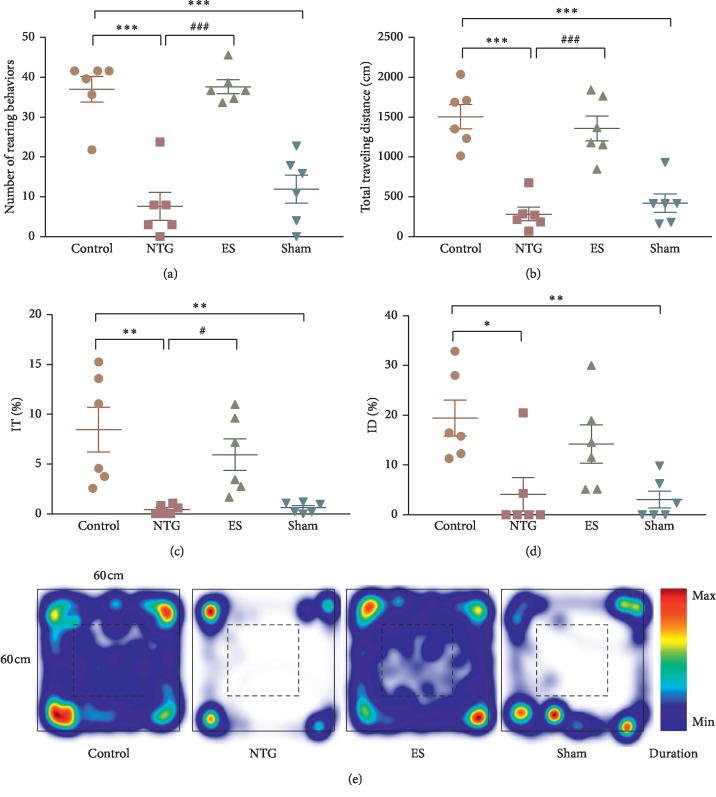
Effects of auricular electrical stimulation (ES) pretreatment on depressive or anxiety-like behaviors in the nitroglycerin (NTG)-induced migraine rat model: (a) frequency of rearing behavior (number/5 min); (b) total distance traveled (cm/5 min); (c) percentage of time spent in the inner zone (IT%); (d) percentage of distance traveled in the inner zone (ID%); (e) group mean heat map plot. Color level of duration: the end colors represent the minimum (dark blue) and the maximum (dark red) occupancy of duration. Control: control group; NTG: NTG group; ES: ES group; sham: sham group. Data are presented as mean ± standard error of the mean with scatter plot. ^*∗*^*p* < 0.05, ^*∗∗*^*p* < 0.01, ^*∗∗∗*^*p* < 0.001 vs control group; ^#^*p* < 0.05, ^##^*p* < 0.01, ^###^*p* < 0.001 vs NTG group.

Depression was assessed on the basis of the frequency of rearing behavior and the total distance traveled. The frequencies of rearing behavior in the NTG (7.7 ± 3.5) and sham (12.0 ± 3.6) groups were lower than that in the control (37.3 ± 3.2) group (both *p* < 0.0001; Figure 3(a)). The frequency of rearing behavior in the ES (38.0 ± 1.8) group was higher than that in the NTG group (*p* < 0.0001; Figure 3(a)).

The distances traveled by the rats in the NTG (285.3 ± 85.6 cm) and sham (423.9 ± 114.7 cm) groups were shorter than that traveled by those in the control (1521.0 ± 154.2 cm) group (both *p* < 0.0001; Figure 3(b)). However, the distance traveled by the rats in the ES (1372.0 ± 157.9 cm) group was longer than that traveled by those in the NTG group (*p* < 0.0001; Figure 3(b)).

Anxiety was assessed on the basis of IT% and ID%. The rats in the NTG (0.4 ± 0.2%) and sham (0.6 ± 0.2%) groups had lower IT% values than did those in the control (8.5 ± 2.3%) group (*p*=0.0028 and *p*=0.0035, respectively; Figure 3(c)), whereas the rats in the ES (6.0 ± 1.6%) group exhibited higher IT% values than did those in the control group (*p*=0.0472; Figure 3(c)).

The NTG (4.2 ± 3.4%) and sham (3.1 ± 1.7%) groups had lower ID% values than did the control (19.7 ± 3.7%) group (*p*=0.0156 and *p*=0.0094, respectively; Figure 3(d)). Nevertheless, no significant difference in ID% was observed between the ES (14.4 ± 3.9%), NTG, and sham groups (all *p* > 0.05; Figure 3(d)).

A plot of mean heat maps for the groups revealed that the rats in the NTG and sham groups intuitively spent more time in the outer zone, especially at the corner, compared with those in the control group. This phenomenon was, however, observed to be similar for the rats in both the ES and control groups (Figure 3(e)).

### 3.3. Effect of Auricular ES Pretreatment on CGRP, COX-2, TRPV1, and TRPA1 Immunoreactive Cells in the TG in NTG-Induced Migraine Rats

**Figure 4:**
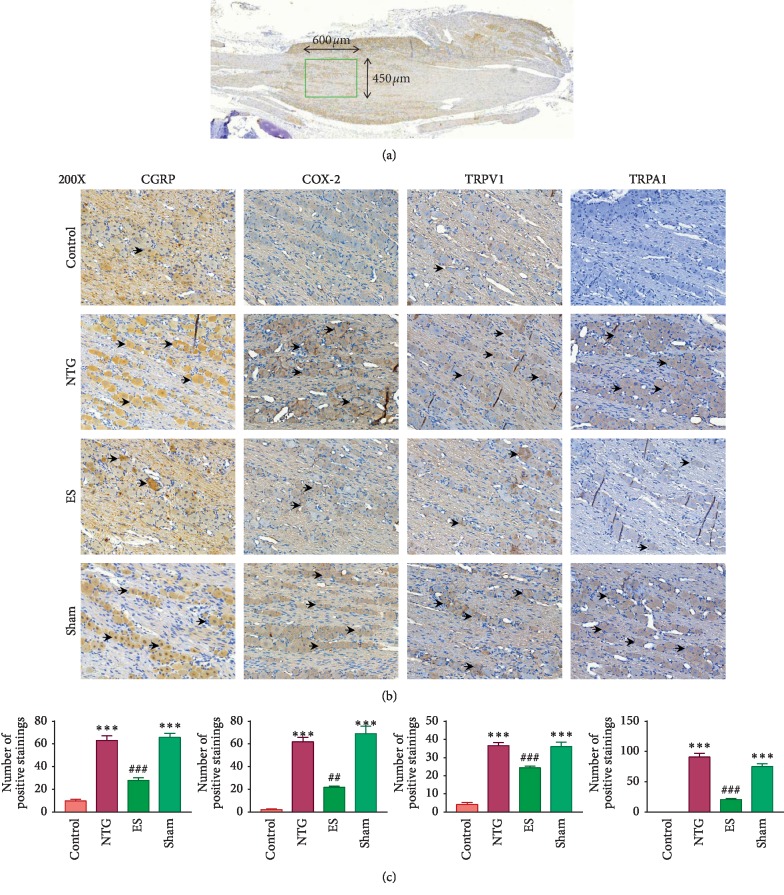
Effects of auricular electrical stimulation (ES) pretreatment on calcitonin gene-related polypeptide (CGRP), cyclooxygenase-2 (COX-2), transient receptor potential cation channel subfamily V member 1 (TRPV1), and (TRPV1), and transient receptor potential cation channel subfamily A member 1 (TRPA1) immunoreactive cells in the trigeminal ganglion (TG) in the nitroglycerin (NTG)-induced migraine rat model: (a) CGRP, COX-2, TRPV1, and TRPA1 immunoreactive cells in the TG (green rectangle); (b) CGRP, COX-2, TRPV1, and TRPA1 immunoreactive cells (arrow head); (c) number of CGRP, COX-2, TRPV1, and TRPA1 immunoreactive cells. Control: control group; NTG: NTG group; ES: ES group; sham: sham group. Data are presented as mean ± standard error of the mean. 200x: magnification of 200x; scale bar = 60 *μ*m; ^*∗*^*p* < 0.05, ^*∗∗*^*p* < 0.01, ^*∗∗∗*^*p* < 0.001 vs control group; ^#^*p* < 0.05, ^##^*p* < 0.01, ^###^*p* < 0.001 vs NTG group.

CGRP immunoreactive cells in the TG of the rats in the NTG (63.0 ± 4.2) and sham (65.8 ± 3.7) groups were increased compared with those in the control (9.7 ± 1.5) group (both *p* < 0.0001; Figures 4(a) and 4(b)). However, this increase was reduced by auricular ES (27.8 ± 2.6) pretreatment (*p* < 0.0001; Figures 4(a) and 4(b)).

The numbers of COX-2 immunoreactive cells in the TG of the rats in the NTG (61.8 ± 4.0) and sham (69.0 ± 6.5) groups were increased compared with those in the control (2.2 ± 0.7) group (both *p* < 0.0001; Figures 4(a) and 4(b)). Nevertheless, this increase was reduced by auricular ES (21.7 ± 1.2) pretreatment (*p* < 0.0001; Figures 4(a) and 4(b)).

The numbers of TRPV1 immunoreactive cells in the TG of the rats in the NTG (36.5 ± 1.7) and sham (36.0 ± 2.4) groups increased compared with those in the control (4.2 ± 1.2) group (both *p* < 0.0001; Figures 4(a) and 4(b)). However, this increase was reduced by auricular ES (24.3 ± 1.0) pretreatment (*p*=0.0003; Figures 4(a) and 4(b)).

Finally, the numbers of TRPA1 immunoreactive cells in the TG of the rats in the NTG (90.8 ± 6.5) and sham (75.0 ± 4.6) groups were increased compared with those in the control (0.0 ± 0.0) group (both *p* < 0.0001; Figures 4(a) and 4(b)). Nevertheless, this increase was reduced by auricular ES (20.7 ± 1.9) pretreatment (*p* < 0.0001; Figures 4(a) and 4(b)).

## 4. Discussion

Auricular acupuncture is a major aspect of clinical acupuncture practice and may play a crucial role in treating migraine. In addition, auricular acupuncture has analgesic and antipsychogenic effects [11]. The results of the present study indicated that auricular ES pretreatment reduced spontaneous nociceptive behaviors and depressive- or anxiety-like behaviors in rats, these results were in good agreement with a randomized controlled trial finding that auricular acupuncture can relieve migraine headache and depression emotion [31], and auricular acupuncture also can prevent the generation of acute and chronic migraine [32].

The objective methods to assess nociceptive pain including mechanical and thermal allodynia in migraine rats or mice include the von Frey test on the periorbital area or plantar surface of the hind paw, and the Hargreaves test [33, 34]. Therefore, the present study was conducted in order to further explain the associated behavior in migraine except nociceptive pain using videotaped recordings analysis. As shown by the results of this study, the total time spent on exploratory and locomotor behaviors decreased but that spent on resting and grooming behaviors increased in rats that received repeated IP injections of NTG. These behaviors are similar to those observed in patients with migraine who typically avoid or reduce the frequency of routine physical activity during a migraine attack [35, 36]. Cutaneous allodynia is a painful sensation resulting from a nonnoxious stimulus to normal skin, which is a symptom frequently associated with migraine attacks [28]. According to a report, approximately 63% of migraineurs present with clinical characteristics of cutaneous allodynia [37]. A longitudinal study revealed that cutaneous allodynia is an independent predictor of chronic migraine [38]. The increase in the total time spent on grooming behaviors by rats is analogous to cutaneous allodynia in patients with migraine. Overall, auricular ES pretreatment is beneficial for the prevention of headaches in patients with migraine.

Psychiatric disorders, especially depression and anxiety, are the most common comorbidities associated with chronic migraine [39, 40]. Several studies have reported that repeated NTG injections might evoke depressive or anxiety-like behaviors or related pathology in rats [41, 42]. To assess depression and anxiety, the present study conducted behavior tests 360 min after IP NTG administration on the fifth day (i.e., during the migraine-free interval). This time point was selected to eliminate the confounding factor of a migraine-like pain attack, which is considered to be present up to 4 h after NTG injection [25, 43]. Open-field tests are highly reliable in testing emotional status in rodents [44]. Notably, patients with migraine and associated depression would often lose interest in activities. Similar to human manifestations, rodents with depression-like emotions exhibited decreased exploration behaviors and had shorter travel distances during the open-field test in this study [30]. When placed in the novel surrounding, the anxious rodents feared to cross the center and tended to stay in a safe place [30]. The results of the present study thus indicate that auricular ES pretreatment could increase the frequency of rearing behavior, the total distance traveled, and the percentage of time spent in the inner zone during the open-field test in rats with NTG-induced migraine; accordingly, the results suggest that auricular ES pretreatment is beneficial for psychological behaviors caused by migraine.

Migraine is a complex disorder, which the pathophysiology was involving in molecules and ion channels, inflammatory pathways, metabolic changes, and biological processes [45]. The CGRP plays a pivotal role in migraine headache [46]. CGRP—a type of excitatory neurotransmitter—is synthesized in the soma of the TG. CGRP is involved in peripheral neurogenic vasodilatation and is considered a causative factor in the pathogenesis of migraine [47]. CGRP receptor antagonists, such as MK-8825 and olcegepant, are used as a potential antimigraine therapy [48, 49]. Notably, central infusion of CGRP could induce poststroke depression in an ischemic rat model, and a CGRP antagonist could produce antidepressant effects [50]. CGRP infused into the lateral ventricle of rats evokes anxiety-like responses, which could be blocked using a CGRP antagonist [51]. COX-2 plays a critical proinflammatory role in pain and inflammation. The expression of COX-2 could be significantly upregulated in the dura mater, TG, and TNC in an NTG-induced migraine model [52, 53]. Hence, COX-2 inhibitors are used to prevent or reduce migraine severity [54]. In addition, recent research demonstrated that COX-2 inhibitors constitute a novel augmentation therapy for stress-related anxiety and depression [55]. The plasma levels of COX-2 are higher in the headache attack period than those in the headache-free period in patients with migraine [56]. The TRPV1 and TRPA1 channels play a critical role in migraine pathophysiology [57, 58]. Both TRPV1 and TRPA1, belonging to the TRP family, are involved in pain modulation. The expression levels of TRPV1 and TRPA1 were elevated in the TNC and TG owing to systemic NTG administration [53, 59]. TRPV1 and TRPA1 also influence emotions. Blockade or deletion of TRPV1 in rodents induced anxiolytic and antidepressant effects [60–62]. Notably, TRPA1 antagonism could also inhibit anxiety and depression in mice [63]. The results of this study indicate that CGRP, COX-2, TRPV1, and TRPA1 immunoreactive cells increased in the TG in the NTG-induced migraine rat model, and these increases were reduced by auricular ES pretreatment. Collectively, the results suggest that auricular ES pretreatment improved both migraine and depressive or anxiety-like emotional behaviors in NTG-induced migraine rats. In addition, auricular ES pretreatment could reduce CGRP, COX-2, TRPV1, and TRPA1 levels.

There are still some limitations in the present study: (1) more objective methods to assess nociceptive pain are needed, such as von Frey test for nociceptive periorbital pain threshold; therefore, more evaluations will be done for assessment of migraine except video recordings for associated behavior of migraine in the future; (2) more objective methods to assess emotional behavior is need, such as forced-swim or tail suspension tests except locomotor activity of open-field test in the future; (3) one experiment is not enough to conclude the signaling pathway; another genetic modification or molecular technique to investigate the signaling is needed in the future; (4) the study must be concerning with the emotional data that can be interrupted by physical factor, which is a locomotor behavior in the future.

In conclusion, auricular ES pretreatment increased the total time spent on exploratory and locomotor behaviors and reduced the total time spent on resting behavior in the NTG-induced migraine rats; these results suggest that auricular ES pretreatment is beneficial in preventing migraine. Auricular ES pretreatment also increased the frequency of rearing behavior, the total distance traveled, and the percentage of total time spent in the inner zone during the open-field test in rats with NTG-induced migraine; these findings indicate that auricular ES pretreatment is helpful in controlling the psychological behaviors caused by migraine. Moreover, this effect of auricular ES pretreatment is partly related to the CGRP/COX-2/TRPV1/TRPA1 signaling pathways.

## Data Availability

The data in this study are available to other researchers upon request.
